# Sensitization to a purified cow’s milk (CM) allergen facilitates further oral sensitization to other CM proteins in mice

**DOI:** 10.1186/2045-7022-1-S1-O29

**Published:** 2011-08-12

**Authors:** Karine Adel-Patient, Hervé Bernard, Sandrine Ah-Leung, Sophie Wavrin, Jean-Michel Wal

**Affiliations:** 1 INRA, Unité d'Immuno-Allergie Alimentaire, Gif-sur-Yvette, France

## Background

the effect of a local allergic reaction induced by a food allergen on the immune response induced against bystander proteins has been not much investigated. We then aimed to compare the effect of oral administrations of raw milk either in naïve mice or in mice previously orally sensitized to bovine β-lactoglobulin (BLG), a major cow's milk allergen.

## Methods

BALB/cJ mice were experimentally sensitized by intra-gastric (i.g.) administration of highly purified BLG mixed with Cholera toxin (CT, 10μg) on days 1, 8, 15, 22, 26 and 33 (n=7/group). Control mice received CT alone (n=7) and 10 mice were left untreated (naïve mice). Allergic sensitization to BLG was confirmed by assaying BLG-specific IgE and IgG1 in sera collected on day 37. Mice administered CT +/- BLG and 5 naive mice were then gavaged on days 39, 40 and 43 to 46 with 200μl of raw CM, naturally containing 3 mg/ml of BLG, without any adjuvant. Antibodies specific to the different CM proteins were then assessed by immunoassays in sera collected on day 54.

## Results

No antibody specific to CM proteins were evidenced in naïve or control mice after gavage with CM. Conversely, mice sensitized to BLG produced specific IgG1 antibodies against caseins (αs1, αs2, β and κ), α-lactalbumin, lactoferrin and bovine serum albumin after gavage with CM.

## Conclusion

BLG present in CM likely elicits a specific local allergic reaction in mice already sensitized to BLG. The resulting pro-Th2 intestinal microenvironment and/or the increase of intestinal mucosa permeability may then favour the induction of a Th2-type response specific to other bystander proteins ingested at the same time. Such mechanism may partly explain multiple sensitizations in food allergic patients.

